# Liver ultrasound evaluation of acutely increased liver function tests of COVID-19 hospitalized patients

**DOI:** 10.5339/qmj.2024.46

**Published:** 2024-09-16

**Authors:** Salah Almughalles, Shazaly N. Khojaly, Abdulqadir J. Nashwan, Adham Darweesh

**Affiliations:** 1 Hamad Medical Corporation, Doha, Qatar; 2 Department of Clinical Imaging, Hamad Medical Corporation, Doha, Qatar *Email: salmughalles@hamad.qa

**Keywords:** COVID-19, hepatic abnormalities, ultrasonography, liver function tests, ACE2 expression

## Abstract

**Background:**

The incidence of hepatic abnormalities has been notably higher following the coronavirus disease 2019 (COVID-19) infection, attributed to the virus’s entry into cells via angiotensin-converting enzyme 2 (ACE2) surface expression. The gastrointestinal tract’s significant ACE2 expression, alongside a lesser degree in the biliary epithelium, has been implicated in gastrointestinal symptoms and liver injury.

**Purpose:**

The aim of this study was to determine whether specific ultrasonographic findings in the liver correlate with acute increases in liver function tests (LFTs) among hospitalized patients.

**Methods:**

A retrospective analysis was conducted on hospitalized COVID-19 patients at Hazem Mebaireek General Hospital in Qatar, from March 1, 2020, to June 30, 2020. The study focused on patients who experienced acute increases in LFTs, excluding those with chronic liver disease. Ultrasound imaging and patient records were reviewed to gather data.

**Results:**

Out of 223 ultrasound studies of COVID-19 patients, 158 met the inclusion criteria. The majority were male, with a mean age of 47.76 ± 13.76 years. Ultrasound results showed 43.7% normal liver parenchyma, while 56.3% exhibited nonspecific abnormalities such as diffuse liver hyperechogenicity (39.2%), enlargement with diffuse hyperechogenicity (12.7%), and other findings (4.4%). The biliary tree was predominantly normal (96.2%), with 3.8% showing abnormalities, including intrahepatic (2.5%) and extrahepatic (1.3%) dilatation. Gallbladder evaluations were normal in 60.1% of cases, with 39.9% showing abnormalities like stones (6.3%), stones with sludge (13.3%), polyps (6.3%), wall thickening (1.9%), and other conditions (12%). A significant correlation was found between abnormal liver parenchyma findings and elevated levels of bilirubin (total and direct) and alkaline phosphatase, with p-values < 0.05. Only aspartate aminotransferase levels showed a significant correlation with biliary tree abnormalities.

**Conclusion:**

The most common ultrasonographic finding associated with acute increases in LFTs among hospitalized COVID-19 patients was diffuse liver hyperechogenicity, with or without enlargement. These findings suggest a nonspecific yet significant association with liver function anomalies in the context of COVID-19.

## 1. Introduction

At the end of 2019, a new coronavirus, severe acute respiratory syndrome coronavirus (SARS-CoV-2), was identified as the culprit behind a series of pneumonia cases in Wuhan, within China’s Hubei Province.^1^ Coronavirus disease 2019 (COVID-19), the disease caused by this virus, predominantly affects the lungs, presenting symptoms that range from mild respiratory issues to severe pneumonia, acute respiratory distress syndrome, and even death.^2^ Healthcare professionals, notably those in gastroenterology and hepatology, are revising their practices to mitigate COVID-19’s spread while continuing to provide patient care.^3^ Although primarily a respiratory illness, COVID-19 also impacts multiple organs, including the heart, kidneys, and liver.^4^ Notably, up to 11% of COVID-19 patients experience liver-related comorbidities, and 14%–53% exhibit elevated levels of liver enzymes (alanine aminotransferase [ALT] and aspartate aminotransferase [AST]) as well as the other parameters of liver function tests (LFTs) such as bilirubin total and direct and alkaline phosphatase (ALP) as the disease progresses, underscoring a significant correlation between the severity of infection and liver enzyme elevation.^5^ Mild cases may show no or slight enzyme elevation, whereas severe cases often have significantly higher levels. Even in mild COVID-19 instances, transient liver damage has been observed, though it typically resolves with minimal treatment.^6^

Diagnostic screening for liver pathology and abnormal LFTs commonly employs ultrasound and evaluations of serum bilirubin and ALP levels.^7^ Liver enzyme disturbances generally manifest in one of two patterns: hepatocellular injury and cholestasis, the former is characterized by disproportionately elevated AST and ALT levels compared to ALP, and the latter is marked by a disproportionate increase in ALP relative to AST and ALT.^7^ Abdominal ultrasound serves as the primary diagnostic tool for liver enzyme abnormalities, complemented by further assessments using computed tomography, magnetic resonance imaging, magnetic resonance cholangiopancreatography, or cholescintigraphy.^7^

The substantial rise in ultrasound requests for liver assessment amid hospitalizations—driven by elevated LFTs and the paucity of comprehensive data on liver ultrasound findings in COVID-19 patients—underscores the urgent need for this study. This research aims to address the existing gap in the literature by delineating specific ultrasound characteristics associated with COVID-19, thereby guiding necessary follow-up and clinical interventions.

## 2. Methods

This retrospective study investigates abdominal ultrasonography examinations conducted on COVID-19 patients hospitalized at the Hazem Mebaireek General Hospital, a primary COVID-19 treatment facility under Hamad Medical Corporation in Qatar. The period of study spanned from March 1, 2020, to June 30, 2020. Liver/Abdomen US studies done during this period were identified from the radiology information system. A total of 223 ultrasonography exams were reviewed. The electronic medical records of the patients who underwent these ultrasound studies were analyzed to identify instances of acutely elevated LFTs during their hospital stay. The selection of cases for study adherence was based on predefined research criteria, aiming to isolate the impact of COVID-19 on liver function as evidenced through ultrasound imaging and LFT results.

### 2.1. Inclusion criteria

- All abdominal ultrasonography US examinations of hospitalized patients with confirmed COVID-19 diagnosis.
- Patients with imaged US exhibited an acute increase in LFTs during their hospitalization period.

### 2.2. Exclusion criteria

- Patients with a negative COVID-19 PCR test result.
- Individuals with a pre-existing history of chronic liver disease, including features of chronic liver conditions or focal lesions on the liver.
- Cases where the ultrasound study was incomplete or lacked sufficient detail for analysis.

Through this methodology, the study aims to identify specific ultrasonographic findings associated with acute liver function alterations in COVID-19 patients, thereby contributing valuable insights into the broader spectrum of the virus’s systemic effects.

### 2.3. Statistical analysis

In this study, statistical analysis was carried out to evaluate the relationship between COVID-19 infection and liver function abnormalities as detected through ultrasound imaging and LFTs. The analysis began with descriptive statistics to summarize the demographic characteristics of the study population, including means and standard deviations for continuous variables like age and LFT levels, and frequencies and percentages for categorical variables such as gender and ultrasound findings. Inferential statistics were then employed to identify any significant associations between the ultrasound findings and elevated LFTs. Chi-square tests were used for categorical variables to compare the prevalence of abnormal ultrasound findings among different patient groups, while t-tests or ANOVA were applied for continuous variables to compare mean LFT levels across groups with varying ultrasound results. A significance level of p < 0.05 was adopted for all tests to determine statistical significance. Additionally, logistic regression analyses may have been conducted to adjust for potential confounders and assess the strength of associations between significant findings and COVID-19 severity, although the specifics of such analyses would depend on the data available, and the hypotheses being tested.

## 3. Results

The study encompassed 158 participants, of whom 151 (95.6%) were male and the remainder were female. The majority, 81 individuals (51.3%), fell within the age range of 41–60 years, with an average age of 47.76 ± 13.76 years (Table 1).

LFTs were extensively analyzed, documenting both the initial elevation levels and the peak levels of LFTs. The mean levels of LFTs among participants are detailed in Table 2. Ultrasound examinations focused on liver parenchyma, which appeared normal in 69 cases (43.7%), although these patients have abnormal LFTs. Notably, among those with abnormal US findings, 39.2% of ultrasounds exhibited diffuse liver hyperechogenicity. The biliary tree was predominantly normal, with exceptions being mild intrahepatic dilatation in four cases (2.5%) and mild extrahepatic dilatation in two cases (1.3%). Gallbladder assessments revealed stones in 10 participants (6.3%) and stones with sludge in 21 participants (13.3%), as summarized in Table 3.

Upon comparing ultrasound findings with LFT results, a significant correlation was observed between liver parenchyma abnormalities and total and direct bilirubin levels (both initial and peak) as well as initial ALP levels, all with p-values < 0.05. Other LFT parameters did not show statistical significance. Similarly, abnormalities in the biliary tree correlated significantly with total and direct bilirubin levels (both initial and peak), with p-values < 0.05, while other parameters were not significantly affected. Gallbladder findings showed significant correlations only with peak ALT and ALP levels (p < 0.05) (Table 4).

Not all patients exhibited elevated LFTs in correlation with ultrasound findings, indicating variability among individuals (Table 5). The most commonly elevated LFT parameter was AST (94.3%), followed by ALT (88%), total bilirubin (57.6%), ALP (51.9%), and direct bilirubin (41.8%). Abnormal total bilirubin was noted in 91 participants, direct bilirubin in 66, ALP in 82, ALT in 139, and AST in 149.

When examining the correlation of abnormal LFTs with gender and age, no statistical significance was found for any LFT parameter across these demographics (Table 6). However, a gender-specific analysis of ultrasound findings indicated a higher prevalence of abnormalities among male patients, though no significant age-related trends were observed, despite the 41–60-year age group being most represented (Table 7).

Finally, a significant association was found between abnormal ultrasound findings and elevated levels of total and direct bilirubin and ALP, with p-values < 0.05. For the biliary tree, only AST levels were significantly correlated (Table 8).

## 4. Discussion

In the context of the ongoing COVID-19 pandemic, there is a pressing need for research into various treatment and intervention strategies. The susceptibility of patients with chronic liver diseases to COVID-19 remains unclear. However, emerging studies suggest that the liver may be vulnerable to coronavirus due to the presence of angiotensin-converting enzyme 2 (ACE2) receptors in the biliary and liver epithelial cells, which serve as binding sites for SARS-CoV-2, potentially leading to organ damage.^8–11^ This connection implies a potential association between liver enzyme levels and SARS-CoV-2 infection. Our research focused on exploring liver pathology and abnormalities in LFTs, using ultrasound imaging as a diagnostic tool in COVID-19 patients. Of the 223 ultrasound examinations reviewed, 158 patients met the inclusion criteria for our study. The findings revealed a predominance of male patients, with an average age of 47.76 ± 13.76 years, aligning with previous research.^12^

Although no statistically significant correlation was found between abnormal LFTs and the age or gender of the participants, it was noted that men were disproportionately affected by the disease, and the severity of the illness increased with age.^12–14^ LFTs, which include measurements of AST and ALT, are crucial for detecting hepatocyte injury. These enzymes are key indicators of liver function and are closely related to the permeability of the hepatocyte membrane.^13^ It is important to note that increased levels of ALT and AST are not exclusive to liver damage and have also been linked to myositis.^15^ Generally, liver failure or impairment in patients with SARS-CoV-2 infection does not correlate with abnormal LFTs, and liver-focused treatments are often not required. The mechanisms underlying abnormal LFTs in COVID-19 patients remain largely undefined.

Our study assessed LFTs for both lower and peak levels, identifying a statistically significant elevation in total bilirubin and ALT among COVID-19 patients, which is consistent with previous findings.^16^ Ultrasound examinations most commonly identified hepatobiliary abnormalities, with 62 of 158 patients displaying diffuse liver hyperechogenicity and 20 of 158 showing an enlarged liver with diffuse hyperechogenicity. These findings are consistent with Abdelmohsen’s 2020 study,^17^ which reported that most cases of hepatomegaly showed elevated LFTs and a bright echo pattern in the hepatic parenchyma. This observation is also detailed in the overview of liver involvement in COVID-19 infection done by Ippolito et al.^18^

The biliary tree appeared normal in the majority of cases. Our sonographic results also highlighted a variety of gallbladder findings, including stones, sludge, luminal mud, mural thickening, polyps, and mural hyperemia in 40.1% of COVID-19 patients, similar to rates observed in both ICU and non-ICU patients in studies by Bhayan et al.^19^ This range of findings suggests that SARS-CoV-2’s attachment to gallbladder epithelial cells may lead to mucosal inflammation, shedding light on the ultrasound observations in COVID-19 patients.^20,21^ When correlating abnormal ultrasound results with LFTs, significant changes were observed in the levels of total bilirubin, ALP, and ALT, indicating that abnormal ultrasound findings could reflect variations in these parameters as the disease progresses, where patients with abnormal US findings correspond to the highest alteration of LFTs. Furthermore, abnormal LFTs’ results may increase the risk of developing severe pneumonia, making LFTs a potential early predictor of disease severity.^22^

### 4.1. Limitations

One of the primary limitations of this study lies in its retrospective design, which inherently restricts the ability to control variables that may influence the outcomes. The reliance on patient records and imaging studies conducted in the past may introduce bias, as the data collected were not initially intended for this research purpose. Furthermore, the study’s focus on a single healthcare facility limits the generalizability of the findings to wider populations, as patient demographics, healthcare practices, and the prevalence of COVID-19 can vary significantly across different regions. Another limitation is the exclusion of patients with pre-existing chronic liver diseases, which could provide valuable insights into how COVID-19 affects individuals with underlying liver conditions. Additionally, the study does not account for the potential impact of concurrent medications or treatments for COVID-19 on LFTs, which could confound the results. Lastly, the absence of a control group of non-COVID-19 patients undergoing similar liver function assessments prevents a direct comparison of ultrasound findings and LFT elevations specifically attributable to COVID-19.

## 5. Conclusion

Ultrasound findings, while helpful, should be interpreted with caution due to their nonspecific nature, as liver parenchyma abnormalities can occur in various conditions, with fatty liver (steatosis) appearance being a common causative feature. The ongoing evolution of COVID-19 underscores the critical need for further research to deepen our understanding of its impact on liver health and the optimal management of patients with liver diseases. Given the common observation of liver impairment in COVID-19 patients, future studies should include comprehensive evaluations, such as serology tests, to better understand the causes of liver failure and impairment. Additionally, as new antiviral vaccines are developed, it may be necessary to update recommendations to ensure their safety and efficacy in patients with liver diseases.

## Conflict of Interest

No conflict of interest to declaire.

## Figures and Tables

**Table 1.** Demographic data of the participants (n = 158).

**Variables**	**Frequency**	**Percentage (%)**
Gender		
Male	151	95.6
Female	07	4.4
Age group		
20–40 years	52	32.9
41–60 years	81	51.3
61–80 years	22	13.9
>80 years	3	1.9
Age mean ± SD	47.76 ± 13.76	

**Table 2.** Liver function tests of the participants (n = 158).

**Variables**	**Mean**	**SD**	**Minimum**	**Maximum**
Bilirubin total lower	29.54	55.58	12	515
Bilirubin total peak	66.55	128.91	21	936
Bilirubin direct lower	29.93	49.43	07	302
Bilirubin direct peak	92.86	129.09	07	582
ALP lower	96.36	59.11	40	306
ALP peak	289.83	344.38	129	1862
ALT lower	82.82	92.01	42	984
ALT peak	513.55	1017.46	55	7000
AST lower	72.31	137.58	36	1360
AST peak	500.33	1017.33	40	7000

Normal range as per HMC core laboratory reference reading with measured units; Bilirubin total umol/L <21, Bilirubin direct umol/L <5, ALP U/L 40–129, ALT U/L <41, AST U/L <40.

**Table 3.** Ultrasound findings of the participants (n = 158).

**Findings**	**Frequency**	**Percentage (%)**
Liver parenchyma		
Normal	69	43.7
Diffuse hyperechogenicity	62	39.2
Enlarge with diffuse hyperechogenicity	20	12.7
Others (coarse or inhomogeneous)	07	4.4
Biliary tree		
Normal	152	96.2
Abnormal intrahepatic	4	2.5
Abnormal extrahepatic	2	1.3
Gall bladder		
Normal	95	60.1
Stone	10	6.3
Stone with sludge	21	13.3
Polyps	10	6.3
Wall thickening	3	1.9
Others (contracted, removed, or non-visualized)	19	12

**Table 4.** Correlation of ultrasound findings with liver function test (n = 158).

	**Bilirubin total lower**	**Bilirubin total peak**	**Bilirubin direct lower**	**Bilirubin direct peak**	**ALP lower**	**ALP peak**	**ALT lower**	**ALT peak**	**AST lower**	**AST peak**
Liver parenchyma’s										
Normal	24.18 ± 9.63	58.73 ± 69.696	21.60 ± 9.49	66.82 ± 64.49	98.57 ± 59.44	236.36 ± 175.21	75.58 ± 68.26	441.76 ± 942.98	73.20 ± 162.40	473.17 ± 1106.41
Diffuse hyperechogenicity	22.63 ± 3.59	57.53 ± 107.174	21.56 ± 11.16	104.97 ± 145.51	81.42 ± 51.98	296.32 ± 418.54	79.38 ± 51.41	565.24 ± 948.85	58.71 ± 24.73	552.08 ± 1075.88
Enlarge with diffuse hyperechogenicity	22.65 ± 4.69	35.98 ± 27.389	19.75 ± 8.24	51.72 ± 29.89	109.93 ± 62.80	398.50 ± 492.59	122.26 ± 209.65	654.77 ± 1572.49	118.37 ± 246.91	486.37 ± 644.48
Others (coarse or inhomogeneous)	163.40 ± 240.20	310.86 ± 431.715	134.0 ± 153.67	276.06 ± 287.36	168.14 ± 49.47	448.86 ± 322.44	75.57 ± 39.96	359.57 ± 238.37	59.57 ± 15.38	332.14 ± 279.26
P-value	<0.001	<0.001	<0.001	0.004	0.001	0.160	0.259	0.800	0.427	0.941
Biliary tree										
Normal	23.29 ± 7.09	54.89 ± 83.80	21.54 ± 10.06	79.3 ± 98.75	94.42 ± 58.45	291.32 ± 350.77	82.83 ± 93.33	512.61 ± 1034.53	72.85 ± 140.32	502.49 ± 1036.1
Abnormal intrahepatic	269.75 ± 283.20	523.75 ± 479.49	159.5 ± 164.5	312.5 ± 311	157.25 ± 28.60	276.0 ± 68.71	62.50 ± 12.34	732.50 ± 460.45	57.25 ± 9.74	622.75 ± 250.52
Abnormal extrahepatic	24.85 ± 5.44	38.0 ± 24.04	24.0 ± 00	38.0 ± 00	122.30 ± 116.39	204.15 ± 106.27	122.55 ± 95.53	145.65 ± 128.19	63.0 ± 32.52	97.0 ± 80.61
P-value	<0.001	<0.001	<0.001	0.001	0.090	0.936	0.755	0.802	0.971	0.832
Gall bladder										
Normal	28.0 ± 50.74	60.79 ± 129.8	29.86 ± 48.56	96.47 ± 148.27	95.11 ± 60.45	275.84 ± 329.63	74.97 ± 36.57	356.63 ± 379.4	56.89 ± 09.99	358.1 ± 552.9
Stone	22.6 ± 1.45	58.8 ± 71.05	26.25 ± 4.64	96.75 ± 77.28	110.4 ± 75.87	273.6 ± 194.24	67.8 ± 16.78	496.8 ± 672.6	71.5 ± 51.9	478.1 ± 840.9
Stone with sludge	22.65 ± 3.96	55.52 ± 44.01	20.58 ± 9.43	61.42 ± 39.96	89.03 ± 55.91	335.2 ± 283.44	123.11 ± 207.7	1243.27 ± 2033.8	103.76 ± 234.9	1083.29 ± 1821.89
Polyps	22.12 ± 1.53	48.32 ± 78.68	18.0 ± 9.89	113.5 ± 125.15	103.34 ± 56.58	155.3 ± 26.94	123.59 ± 166.03	195.39 ± 265.26	196.78 ± 436.23	316.11 ± 636.7
Wall thickening	24.33 ± 4.04	82.00 ± 45.03	15.2 ± 0.00	86.0 ± 0.00	137.33 ± 12.70	1212 ± 805.4	75.67 ± 4.61	301.67 ± 63.50	48.67 ± 6.35	620.33 ± 31.75
Others (contracted, removed, or non-visualized)	53.27 ± 112.9	118.74 ± 213.1	46.56 ± 85.23	111.73 ± 163.49	93.16 ± 54.08	243.32 ± 233.90	63.86 ± 11.35	676.58 ± 1561.0	56.63 ± 11.57	617.26 ± 1567.24
P-values	0.523	0.594	0.861	0.964	0.772	<0.001	0.169	0.011	0.074	0.096

Normal range as per HMC core laboratory reference reading with measured units; Bilirubin total umol/L <21, Bilirubin direct umol/L <5, ALP U/L 40–129, ALT U/L <41, AST U/L <40.

**Table 5.** Percentage of normal to abnormal LFTs.

**Liver function test**	**Normal**	**Abnormal**
Bilirubin total	67 (42.4%)	91 (57.6%)
Bilirubin direct	92 (58.2%)	66 (41.8%)
ALP	76 (48.1%)	82 (51.9%)
ALT	19 (12.0%)	139 (88.0%)
AST	9 (5.7%)	149 (94.3%)

**Table 6.** Correlation of age and gender with abnormal LFTs.

	**Bilirubin total (n = 91)**	**Bilirubin direct (n = 66)**	**ALP (n = 82)**	**ALT (n = 139)**	**AST (n = 149)**
Gender					
Male	86	62	76	134	142
Female	05	4	06	05	07
P-value	0.365	0.322	0.071	0.199	0.65
Age group					
20–40 years	29	18	23	42	49
41–60 years	45	34	44	74	75
61–80 years	14	11	14	21	22
>80 years	03	3	1	02	03
P-value	0.284	0.044	0.230	0.165	0.439

**Table 7.** Correlation of age and gender with ultrasound findings.

	**Gender**			**Age group**		
Liver parenchyma’s	Male	Female	20–40	41–60	61–80	>80
Normal	65	4	22	36	9	2
Diffuse hyperechogenicity	61	1	18	32	11	1
Enlarge with diffuse hyperechogenicity	20	0	7	13	0	0
Others (coarse or inhomogeneous)	05	2	5	0	2	0
P-value	0.007			0.150		
Biliary tree						
Normal	145	7	49	78	22	3
Abnormal intrahepatic	4	0	2	2	0	0
Abnormal extrahepatic	2	0	1	1	0	0
P-value	0.865			0.957		
Gall bladder						
Normal	92	3	36	47	10	2
Stone	10	0	2	5	3	0
Stone with sludge	18	3	4	10	6	1
Polyps	10	0	4	5	1	0
Wall thickening	3	0	2	1	0	0
Others (contracted, removed, or non-visualized)	18	1	4	13	2	0
P-value	0.28			0.515		

**Table 8.** Correlation of abnormal ultrasound findings with abnormal LFTs.

	**Bilirubin total**	**Bilirubin direct**	**ALP**	**ALT**	**AST**
Liver parenchyma’s					
Diffuse hyperechogenicity	28	20	25	58	60
Enlarge with diffuse hyperechogenicity	11	6	12	18	18
others	06	5	7	06	07
P-value	0.036	0.041	0.002	0.283	0.547
Biliary tree					
Abnormal intrahepatic	4	4	4	4	4
Abnormal extrahepatic	1	1	1	1	1
P-value	0.217	0.055	0.149	0.194	0.022
Gall bladder					
Stone	5	6	5	9	10
Stone with sludge	14	9	10	17	20
Polyps	4	8	6	9	9
Wall thickening	3	1	3	3	3
Others	13	8	10	18	19
P-value	0.342	0.171	0.656	0.80	0.730
